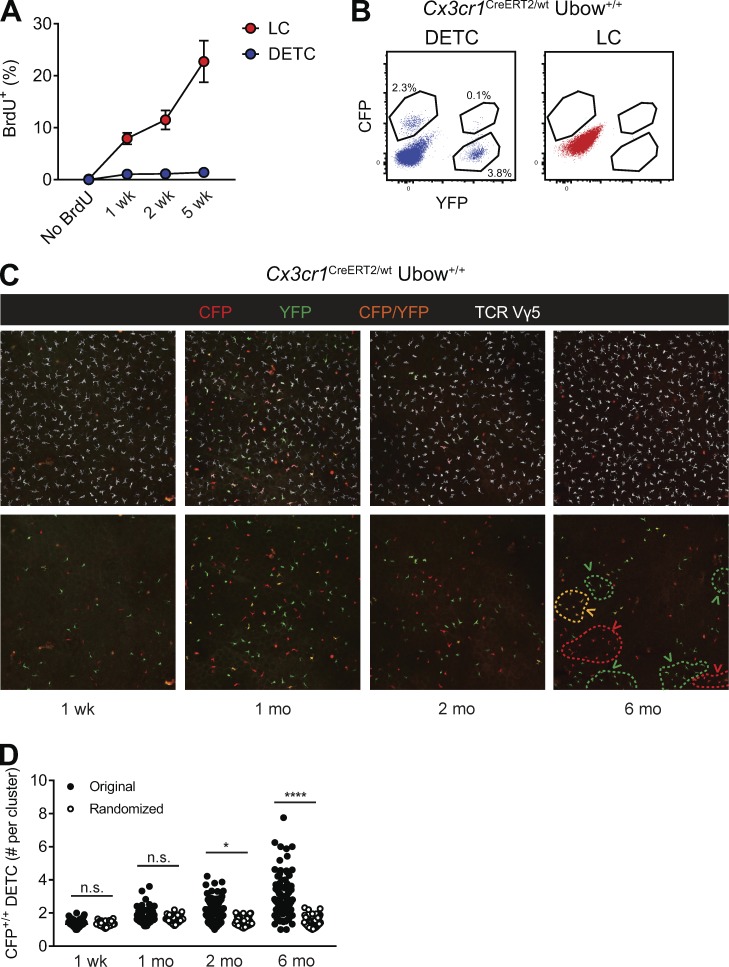# Correction: Epidermal γδ T cells originate from yolk sac hematopoiesis and clonally self-renew in the adult

**DOI:** 10.1084/jem.2018120611142018c

**Published:** 2018-12-03

**Authors:** Rebecca Gentek, Clément Ghigo, Guillaume Hoeffel, Audrey Jorquera, Rasha Msallam, Stephan Wienert, Frederick Klauschen, Florent Ginhoux, Marc Bajénoff

Vol. 215, No. 12, December 3, 2018. 10.1084/jem.20181206.

*JEM* regrets that in the original version of this paper, the labels in Fig. 2, C and D, appeared incorrectly as a result of a production error. The figure appears below with the corrected labels “1 mo,” “2 mo,” and “6 mo.” All versions of this article have been corrected.

**Figure:**